# Morpho‐physiological and agronomic responses of wheat varieties under artificial shade in agroforestry systems

**DOI:** 10.1002/jsfa.70157

**Published:** 2025-09-04

**Authors:** Anna Panozzo, Pranay Kumar Bolla, Giuseppe Barion, Giovanna Visioli, Teofilo Vamerali

**Affiliations:** ^1^ Department of Agronomy, Food, Natural Resources, Animals and the Environment University of Padua Padua Italy; ^2^ Department of Chemistry, Life Sciences and Environmental Sustainability University of Parma Parma Italy

**Keywords:** leaf greenness, grain protein, old wheat varieties, shade tolerance, silvoarable systems

## Abstract

**BACKGROUND:**

Solar radiation is a primary constraint in silvoarable agroforestry, with yield losses near the trees well documented in temperate climates. However, genetic variability for shade tolerance remains largely unexplored. This 2‐year field trial investigated the impact of artificial shading – using nets that reduced photosynthetically active radiation (PAR) by moderate (−30%) and severe (−50%) levels relative to full sun – on the morpho‐physiology and yield of common wheat. During the first growing season, three varieties were evaluated: the modern variety, *Bologna,* and two old, tall varieties, *Piave* and *Terminillo*. In the second season, only *Bologna* was retested under the same shading treatments, to further assess its performance.

**RESULTS:**

Shading treatments delayed crop phenology from heading onward. They were associated with a prolonged canopy stay‐green, with effects accentuated by increasing shading severity. In the first year, moderate shading increased *Bologna*'s grain yield by 8% (*P* ≤ 0.05) and raised protein content by 0.7%. In the second year, drier conditions and reduced radiation from March–June – causing more severe limitation of PAR, the amount of sunlight available for photosynthesis – led to a 40% yield loss. Old varieties suffered yield reductions due to lodging (−43% in *Terminillo,* −66% in *Piave*), yet showed notable increases in mineral content (up to +48% Mg in *Piave*) and grain protein (+1.7% in *Terminillo* and +3.4% in *Piave*). Under shaded conditions, increased gliadin content led to enhanced gluten accumulation, potentially improving dough extensibility.

**CONCLUSIONS:**

Although nets can mimic shading in wheat effectively, these results highlight the need to extend varietal screening to identify suitable genotypes and key morpho‐physiological traits to optimize wheat cultivation in shaded environments such as agroforestry systems, with further validation in specific emerging models. © 2025 The Author(s). *Journal of the Science of Food and Agriculture* published by John Wiley & Sons Ltd on behalf of Society of Chemical Industry.

## INTRODUCTION

Rising temperatures and changing rainfall patterns due to climate change are affecting the phenology and yield of key staple crops, including wheat. Temperature increases have the most pronounced influence on plant development. However, temperature variations tend to be less severe under shaded conditions in open‐field environments. Elevated temperatures accelerate senescence and shorten the wheat growing cycle.^1^, ^2^ Both vegetative and reproductive phases are shortened due to early flowering and maturity,^3^ and the frequency of heat stress during grain filling has increased in recent decades, posing an increasing threat to yield stability.

Deforestation and the expansion of arable land alone will be insufficient to meet food demands for a growing population.^4^ To address this challenge, farming systems must become more resilient, for instance by integrating mixed tree‐crop cultivation techniques.^5^

Agroforestry systems integrate woody species with crops grown beneath or between the trees, fostering beneficial ecological interactions.^6^, ^7^ Studies highlight the role of agroforestry in enhancing crop tolerance to climate stress through improved resource‐use efficiency and microclimate regulation, including shading and wind protection.^8^, ^9^, ^10^ They mitigate extreme temperatures near trees and reduce evapotranspiration.^11^, ^12^ Despite these benefits, agroforestry remains underutilized in European agriculture covering only 358 000 ha.^13^ A major constraint on wider adoption is the limited knowledge of the performance of crop species and varieties when intercropped with trees.

Among environmental factors, solar radiation is the primary limiting resource for the understorey crops in silvoarable systems.^14^, ^15^, ^16^ Winter cereals, like wheat and barley, are well suited to these systems,^17^ due to their C3 photosynthesis, lower light saturation point, and minimal overlap with the leafing period of deciduous trees. However, the effect of shading on wheat grain yield remains controversial. Some studies reported yield benefits with slight reductions in radiation,^18^, ^19^, ^20^, ^21^, ^22^ whereas others observed yield losses in low irradiance environments from both tree‐based shading^15^ and artificial shading.^23^, ^24^, ^25^ Wheat responses to low light levels are influenced strongly by shading intensity and the phenological stage at which shading occurs. Light distribution is further affected by plant and canopy architecture; for instance, Burgess *et al*.^26^ showed that uneven light exposure can induce photoinhibition, reducing cumulative carbon assimilation in wheat under field conditions. Variety selection might also be relevant, as current breeding is typically conducted under full‐light conditions. Many studies therefore emphasize the need to develop breeding programs targeting shade tolerance in field crops.^27^, ^28^ Meanwhile, there is a need to investigate both the intraspecific and the interspecific variability of crop species and identify traits that confer adaptation to agroforestry farming systems.^18^, ^29^

Within this framework, the present study investigated the impact of shading on winter wheat by using shading nets to simulate agroforestry conditions. Two levels of shading, namely moderate (−30%) and severe (−50%) photosynthetically active radiation (PAR) reductions, were applied from stem elongation to maturity and were compared with full sun controls. The study evaluated a modern variety (*Bologna*) alongside two old, tall varieties (*Piave* and *Terminillo*) to (i) assess varietal response/tolerance to shading in terms of yield, (ii) identify key morpho‐physiological traits associated with shade tolerance, and (iii) examine possible interactions with plant mineral nutrition.

## MATERIALS AND METHODS

### Experimental design

A field trial was conducted during the 2018–2019 and 2019–2020 growing seasons at the Lucio Toniolo experimental farm of the University of Padua, Italy (Legnaro, Padua, Northeast Italy). The site has a silty‐loam soil (US Department of Agriculture classification: Fulvi‐Calcaric Cambisol), comprising 19% clay, 65% silt, 16% sand, 1.65% organic matter, 0.1% total N content, a cation‐exchange capacity (CEC) of 11.4 cmol (+) kg^−1^, and a pH 7.75. Three common wheat varieties (*Triticum aestivum* L.) were evaluated: *Bologna* (SIS, Bologna, Italy), which is a modern hard variety, and two old, tall local varieties, *Piave* and *Terminillo*, preserved at the ‘N. Strampelli’ Institute of Genetic and Agricultural Research in Lonigo (Vicenza, Northeast Italy).

In the first year, sowing took place on 25 October 2018 at a rate of 228 kg ha^−1^ for *Bologna* and 60 kg ha^−1^ for *Piave* and *Terminillo*, with rows spaced 12.5 cm apart. The different seeding rates reflect the high tillering ability of the old varieties, and the need to reduce their lodging due to high culm size. Based on the positive outcome for the *Bologna* variety under shading in the first year, the second‐year experiment focused on this modern variety only to further evaluate its performance under different environmental conditions, by sowing it on 31 October 2019 at the same seed density as the first year.

In both years, the soil was ploughed to a depth of 0.3 m and, before sowing, fertilized with 32 kg ha^−1^ of N, 96 kg ha^−1^ of P_2_O_5_, and 96 kg ha^−1^ of K_2_O, incorporated in the soil by harrowing at a depth of 0.15 m. The previous crop was sugar beet. Seeds were treated with Redigo fungicide (Bayer CropScience, Monheim am Rhein, Germany), containing Prothioconazole and Tebuconazole. From tillering to stem elongation, N was applied twice as granular ammonium nitrate (26.5% N), with an additional foliar application at anthesis, giving a total of 160 kg N ha^−1^ for the *Bologna* variety for both years. Based on preliminary trials, the *Piave* and *Terminillo* varieties received two soil applications of N at the same time as *Bologna*, but at halved doses, totaling 92 kg N ha^−1^. Fungal pathogens were controlled by spraying Azoxystrobin and Cyproconazole twice (mid‐April and mid‐May) in both 2019 and 2020. Manual harvests were conducted on 26 June 2019 and 29 June 2020, respectively.

Artificial shading was applied to 4 m^2^ area wheat plots using white polyvinyl chloride (PVC) nets (Pagini AGV, Pianiga, Venice, Italy) mounted on 2 m high steel cubic frames from 19 April onwards in both years. This time corresponds to the onset of poplar leaf growth in NE Italy, which aligns with the wheat stem elongation stage, typically occurring during mid‐April. Although extending the shading period from sowing might simulate continuous canopy cover, field data indicate that considerable shading in poplar‐based agroforestry systems occurs after leaf development, whereas wheat growth during winter is negligible. Thus, the chosen period adequately depicts canopy development seen in poplar stands. The selected shading levels (30% and 50% PAR reduction), confirmed by periodic measurements beneath the nets relative to full‐sun conditions – were selected to represent the typical overall range of light attenuation observed in mature tree‐based silvoarable systems with narrow inter‐row spacing, and in intensive agrivoltaic systems, without reference to a specific canopy configuration.^30^, ^31^ The 2 m frames provided sufficient vertical clearance for the development of even the old, tall varieties, while mimicking the light interception from growing tree canopies. The experiment followed a completely randomized experimental design, with three wheat varieties, each subjected to two shading levels, −30 and −50% PAR reduction versus full sun controls (C) in an adjacent area, with three replicates.

### Climatic conditions during the experiment

During the first growing season (2018–2019), the average monthly temperature was approximately 1 °C lower than the 10‐year historical mean in December, January, and April, and as much as −3.5 °C lower in May (14.8 °C vs 18.3 °C). In the other months, there were slight increases (<1 °C), except for a warmer June (+2.7 °C) (Supporting Information, Fig. SI.1). Total precipitation from October 2018 to June 2019 was 617 mm, 54% (331 mm), falling between April and May. The winter months (November–March) were drier, recording only 134 mm in comparison with an historical 333 mm.

In the second growing season (October 2019–June 2020), average monthly temperatures were +1 °C higher than the historical mean during the winter months from October to February and similar to the reference in other months. Precipitation varied, with higher amounts in November and December (240 vs 150 mm) after wheat emergence, and markedly lower rainfall from January to May (134 mm versus 335 mm historical). Thus, the two growing seasons contrasted substantially, with the second being much drier.

### Wheat phenology

The Badische Anilin‐ und Soda Fabrik (BASF), Bayer, Ciba‐Geigy, and Hoechst (BBCH) scale was used to record wheat phenological stages.^32^ The BBCH stage of each ‘variety × replicate’ was recorded on two dates during the heading‐flowering period, 2 and 7 May in 2019, and 24 April and 4 May in 2020.

### Vegetation indices

From the last week of April until maturity in June, the normalized difference vegetation index (NDVI) was measured twice weekly on each plot replicate using an active handheld Greenseeker spectrometer (Ntech Industries, Ukiah, CA, USA). The sensor, held approximately 50 cm above the canopy, recorded canopy reflectance at 590 nm red and 880 nm near infrared (NIR), and calculated the index using the standard formula:

$$\text{NDVI}=\frac{\text{refNIR}-\text{refRED}}{\text{refNIR}+\text{refRED}}$$

This index, which ranges from 0 to +1, accurately indicates canopy leaf greenness of the crop together with plant health and coverage of the soil by the vegetation.

The leaf chlorophyll content was indirectly and non‐destructively assessed as a leaf greenness index using a SPAD‐502 chlorophyll meter (Konica Minolta, Hong Kong),^33^, ^34^ serving as a rapid proxy for relative chlorophyll level and overall leaf health. Measurements were taken once during the last week of May (a month after net application) on the flag leaves of six randomly selected tagged plants per plot. Two readings per leaf (at one‐third and at two‐thirds of the leaf length) were recorded and averaged.

### Shoot morphological traits

In the second year (2019–2020), morphological traits were assessed only in the *Bologna* variety to evaluate its growth under the specific environmental conditions of that season with varying shading. The leaf area index (LAI) and the culm area index (CAI) were determined on 14 June, corresponding to maximum leaf expansion and before senescence, using a LI‐3100 leaf area meter (Li‐Cor Inc., Lincoln, Nebraska, USA). Wheat plants were sampled from a 1 m row positioned at the center of the 4 m^2^ plots, away from the borders to avoid edge effects. Leaves were separated from culms and scanned to calculate the leaf‐to‐culm area ratio (LAI/CAI). To capture the full canopy profile and its impact on light interception, six randomly selected plants per plot were also measured for plant height (from coleoptile base to awn tip, including spike), spike length (excluding awns), and the last internode length (from the flag leaf to the spike base).

### Grain yield and quality

Grain yield was measured at maturity by manually harvesting all plants from a 2 m^2^ central area for each plot/replicate (*n* = 3), and threshing with a stationary thresher. The harvest index (HI), which is the grain‐to‐total‐shoot weight ratio, was determined from a 0.5 m^2^ sample from each plot, after oven drying at 105 °C for 36 h. Testing weight (weight of 1 hL of grains) was measured with the portable grain tester GAC 500XT (Dickey‐John, Auburn, IL, USA), and three samples of 1000 grains per variety/treatment/replicate were used to calculate the thousand kernel weight (TKW). Plant biomass at harvest (dry weight) was determined after oven drying for 48 h at 65 °C.

In both years, grain and straw nitrogen concentrations were determined using the Kjeldahl method,^35^ and P, Ca, Mg, and K concentrations were measured using inductively coupled plasma‐optical emission spectroscopy (ICP‐OES) (SPECTRO CirOS Vision EOP; SPECTRO Analytical Instruments GmbH, Kleve, Germany). Samples of 0.4 g were microwave acid‐digested with 7 mL HNO_3_ (65% v/v) and 1 mL H_2_O_2_ (30% v/v) using the high‐performance microwave digestion system ETHOS 900 (Milestone, Bergamo, Italy) following US Environmental Protection Agency (EPA) method 3052.^36^ Analytical accuracy was verified using certified reference materials (ERM‐CD281 and BRC‐402; JRC‐IRMM, Geel, Belgium).

### Grain protein content and gluten composition

Gluten proteins were analyzed on 30 g kernel samples (*n* = 3) milled gently with six 10 s pulses using a Knifetec 1095 (Foss, Hillerod, Denmark). Gliadins, high‐molecular‐weight glutenin subunits (HMW‐GS), and low‐molecular‐weight glutenin subunits (LMW‐GS) were sequentially extracted from 30 mg subsamples, following the protocol described by Visioli *et al*.^37^ Relative quantification of HMW‐GS, LMW‐GS, and gliadins was performed spectrophotometrically using the Bradford colorimetric assay (Bio‐Rad, Hercules, CA, USA) at a 595 nm wavelength, with three technical replicates per sample. Calibration with bovine serum albumin (BSA) standards provided a linear regression between absorbance and protein concentration, with results expressed as mg g^−1^ of wheat flour.

### Statistical analysis

Data on plant morphology, yield and its components, and grain quality parameters were processed using analysis of variance (ANOVA) on R studio software (ver. 1.4; RStudio Public Benefit Corporation (PBC), Boston, MA, USA). In the first growing season (2018–2019), a two‐way ANOVA was performed with ‘variety’, ‘shading treatment’, and their interaction as fixed factors:

Y=μ+variety+shading+variety×shading+ε

where ***Y*** is the response variable, **μ** is the overall mean, and *ε* is the residual error. In the second season (2019–2020), a one‐way ANOVA was performed with ‘shading treatment’ as the only factor:

Y=μ+shading+ε

Means were separated using the Tukey HSD test at *P* ≤ 0.05. Before conducting post hoc comparisons, all data were first subjected to ANOVA to determine the significance of the main effects and interactions.

Principal component analysis (PCA) and multigroup discriminant analysis (MDA), were performed, using Wilks' lambda and Pillai's trace tests,^38^ in MS Excel XLSTAT (Addinsoft, Paris, France) to describe the overall wheat response to artificial shading level as a function of variety choice and year of trial. Before analysis, multivariate normality was assessed using the Shapiro test (in R 3.0.1),^39^ and variables were standardized by subtracting the mean and dividing by the standard deviation.

## RESULTS

### Wheat phenological responses to shading

**Figure 1:**
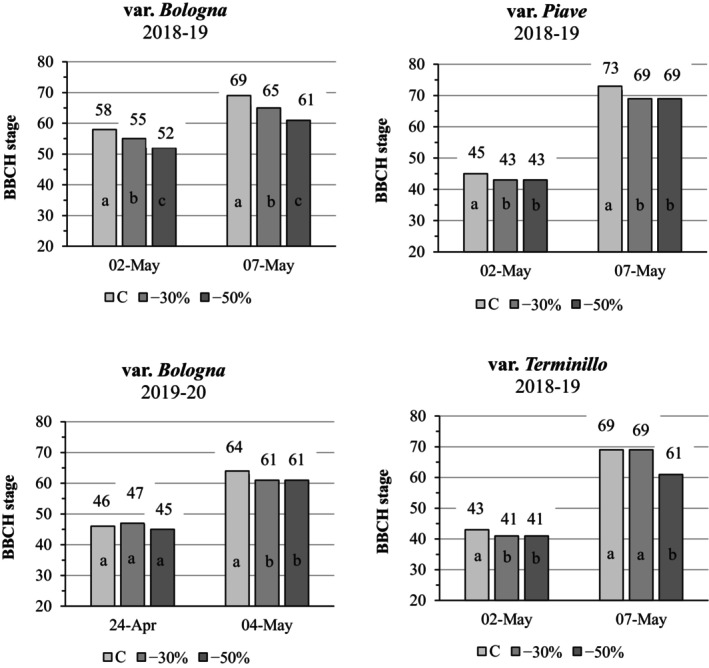
Phenological stage of wheat, according to the BBCH scale,^32^ for *Bologna* (2018–2019 and 2019–2020) and *Piave* and *Terminillo* (2018–2019 only) at two observation dates under two artificial shading treatments (−30 and −50% photosynthetic active radiation (PAR)) versus full sun controls (C).

Wheat heading and flowering dates, assessed using the BBCH scale, differed with variety and year. In the first year, shaded treatments of the *Bologna* variety showed a significant phenological delay at both observation dates, proportional to shade level (approximately 3 days at −30% PAR and 5 days at −50% PAR) compared with full‐sun controls (Fig. 1). In contrast, the old varieties showed smaller delays (~2 days) for both the end of heading (BBCH 59) and complete flowering (BBCH 69), although the *Terminillo* variety exhibited a greater delay on 7 May under severe shading conditions. In the second year, the effect of shading on the *Bologna* variety was less evident and did not follow a strict proportional relationship with shade intensity; however, at the second observation date (4 May 2020), plants still showed a significant phenological delay in both the shaded treatments (BBCH 61 vs BBCH 64 in controls). Given these minimal shading‐induced delays, differences among treatments in other morphological traits are likely to be small.

### Vegetation indices and leaf greenness under increased shading versus full sun

**Figure 2:**
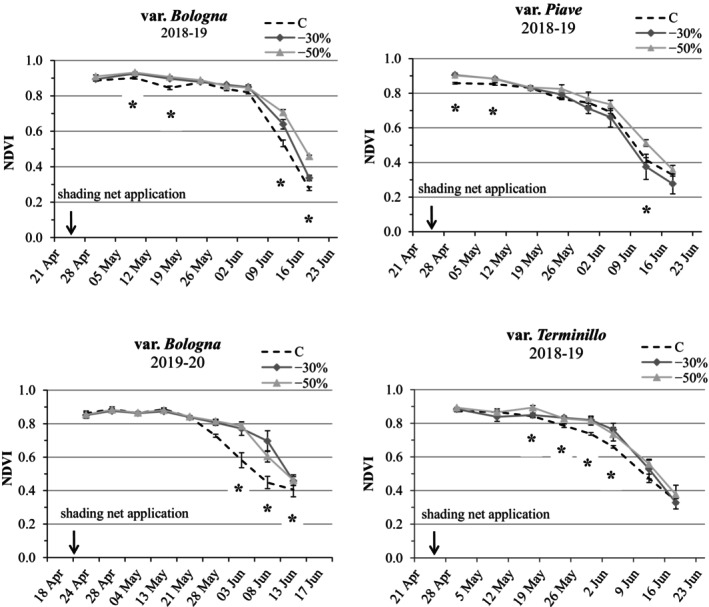
Dynamics of the normalized difference vegetation index (NDVI) from stem elongation to maturity (mean ± SE; *n* = 3) for the *Bologna* variety (2018–2019 and 2019–2020) and *Piave* and *Terminillo* (2018–2019 only), under two artificial shading treatments (−30 and −50% photosynthetic active radiation (PAR)) versus full sun controls (C). Asterisks indicate significant differences among treatments within the same observation time (Tukey's honestly significant difference (HSD) test, *P* ≤ 0.05).

In the first growing season, shaded treatments generally recorded higher NDVI values than controls for the *Bologna* variety, the NDVI increase being significant during heading‐flowering in May (+3% vs C for both −30 and −50% PAR treatments) and during senescence in June; the increases were almost proportional to shading (+21% at −30% PAR and +50% at −50% PAR vs C) (Fig. 2; Supporting Information, Table SI.1). In the *Terminillo* variety, shaded treatments also enhanced NDVI (+7% on average vs C) during May, whereas in the *Piave* variety only the severe shading treatment resulted in a significantly improved NDVI (approximately +13% on average in comparison with C).

In the second growing season, significant NDVI increases under shading treatments were again recorded in the *Bologna* variety from late May to mid‐June, with improvements ranging from +12 to +45% (*P* ≤ 0.05), depending on the observation date. In particular, on 8 June 2020, moderate shading resulted in a +55% increase in NDVI in comparison with +35% under severe shading relative to controls, as visually observed (Supporting Information, Fig. SI.3).

**Figure 3:**
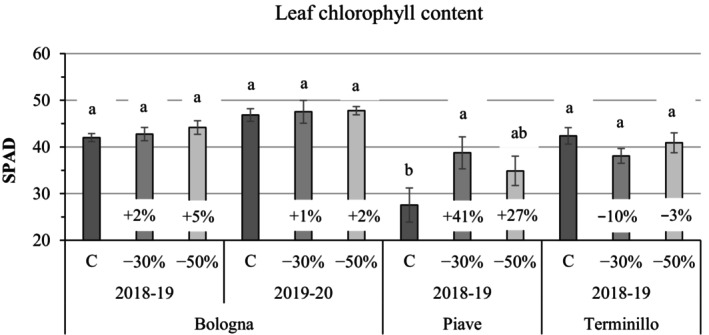
Flag leaf chlorophyll content (SPAD, soil plant analysis development) (mean ± SE; *n* = 3) measured during the last week of May for the *Bologna* variety (2018–2019 and 2019–2020) and the *Piave* and *Terminillo* varieties (2018–2019 only), under two shading treatments – −30 and −50% photosynthetic active radiation (PAR) – versus full sun controls (C). Within each variety and year, numbers above histograms indicate the percentage variation of the shading treatment relative to the control, and different letters indicate statistically significant differences among treatments (Tukey's honestly significant difference (HSD) test, *P* ≤ 0.05).

The leaf chlorophyll content soil plant analysis development (SPAD) values, measured on the flag leaf a month after the application of the shading nets (late May), showed trends similar to NDVI in the *Bologna* variety, with slightly higher SPAD values under shading, particularly under severe shading conditions (−50% PAR). In contrast, the two old varieties displayed more variable responses: *Piave* demonstrated a significant increase in SPAD (+41% versus controls at −30% PAR, *P* ≤ 0.05), whereas *Terminillo* experienced a decrease (approximately −10% vs controls under moderate shading, *P* ≥ 0.05) (Fig. 3; Supporting Information, Table SI.1).

### Shoot morphological traits of the *Bologna* variety

In the second year, shading in the *Bologna* variety reduced LAI (−9% at −30% PAR and −15% at −50% PAR vs controls) but increased the LAI‐to‐CAI ratio (+6% at −30% and +11% at −50% PAR), although these changes were not significant (Supporting Information, Table SI.2). Other morphological traits showed only minor, non‐significant variations, including a slight reduction in plant height (−2% at −30% PAR vs. controls) and a tendency for increased spike length under severe shading (up to +5% vs controls). The last internode length increased slightly under both shading levels, particularly at −30% PAR (+7% vs controls, *P* ≥ 0.05).

### Grain yield and its components under shading

The *Bologna* variety exhibited a different response to shading across the two seasons. In the first year, grain yield improved slightly under moderate shading (+8% vs C, *P* ≥ 0.05) but was significantly reduced under severe shading (*−*19%). In the second year, both shading treatments resulted in considerable yield reductions, particularly under moderate shading (−55% vs C; *P* ≤ 0.05) (Table 1). The old *Piave* variety experienced a marked yield impairment irrespective of shading intensity (about *−*66% vs C), and *Terminillo* suffered yield losses of 43% in comparison with C under moderate shading (not statistically significant).

**Table 1:** Plant biomass, grain yield, and its components (means ± SE; *n* = 3) for *Bologna* (2018–2019 and 2019–2020), *Piave* and *Terminillo* (2018–2019 only), under two shading treatments (−30 and −50% PAR) compared with full sun controls (C).

Variety	Year	Treatment	Yield (g DW m^−2^)		Plant biomass (kg DW m^−2^)		Harvest index (HI)		Testing weight (kg L^−1^)	
*Bologna*	2018–2019	C	675 ± 20a	Ref.	2.03 ± 0.03 a	Ref.	0.36 ± 0.02 a	Ref.	79.8 ± 0.3 a	Ref.
		−30% PAR	730 ± 19a	(+8%)	1.99 ± 0.03 a	(−2%)	0.37 ± 0.01 a	(+1%)	78.2 ± 0.2 b	(−2%)
		−50% PAR	550 ± 20b	(−19%)	1.74 ± 0.06 b	(−14%)	0.32 ± 0.01 a	(−13%)	80.0 ± 0.4 a	(+0.3%)
	2019–2020	C	840 ± 55a	Ref.	1.96 ± 0.11 a	Ref.	0.38 ± 0.01 a	Ref.	78.8 ± 0.5 a	Ref.
		−30% PAR	375 ± 29b	(−55%)	1.55 ± 0.08 b	(−21%)	0.21 ± 0.02 b	(−43%)	75.3 ± 0.8 a	(−4%)
		−50% PAR	522 ± 125ab	(−38%)	1.59 ± 0.12 b	(−19%)	0.25 ± 0.04 ab	(−33%)	76.7 ± 1.1 a	(−3%)
*Piave*	2018–2019	C	336 ± 62a	Ref.	1.57 ± 0.11 a	Ref.	0.21 ± 0.03 a	Ref.		
		−30% PAR	114 ± 47b	(−66%)	1.30 ± 0.05 b	(−17)	0.09 ± 0.03 b	(−59%)		
		−50% PAR	111 ± 18b	(−67%)	1.30 ± 0.05 b	(−17%)	0.09 ± 0.02 b	(−59%)		
*Terminillo*	2018–2019	C	238 ± 49a	Ref.	1.58 ± 0.17 a	Ref.	0.15 ± 0.02 a	Ref.		
		−30% PAR	136 ± 52a	(−43%)	1.36 ± 0.18 a	(−14%)	0.09 ± 0.03 a	(−37%)		
		−50% PAR	242 ± 65a	(+2%)	1.60 ± 0.15 a	(+2%)	0.15 ± 0.03 a	(−1%)		

	Year	Factor				Significance levels				
ANOVA	2018–2019	Variety	***		***		***			
		Shading	*		n.s.		*		*	
		Variety × shading	**		n.s.		*			
	2019–2020	Shading	*		*		*		n.s.	

For each parameter, different letters indicate statistically significant differences among treatments (Tukey's HSD test, *P* ≤ 0.05) within each variety and year, and the values in parentheses represent the percentage variation of the shading treatment relative to the control (Ref.). Results of ANOVA are reported in the bottom block, with significance levels for the main effects (Variety and Shading) and their interaction (n.s. = not significant; *, ** and *** = indicate significance at *P* ≤ 0.05, *P* ≤ 0.01, and *P* ≤ 0.001, respectively). Abbreviation: HI, harvest index.

Testing weight showed slight negative variations (up to −4%) under shading versus C for the *Bologna* variety, whereas the TKW was either slightly improved (in the *Bologna* variety) or reduced (in the old varieties), with a reduction of −15% in comparison with C in *Piave* (Table 1).

In the *Bologna* variety, plant biomass and HI were significantly reduced under both shading intensities in the second year (−20 and −37% respectively vs controls), and under severe shading only in the first year. The *Piave* variety showed significantly reduced plant biomass (*−*17%) and HI (*−*59%) under both shading treatments, whereas *Terminillo* was impacted only under the severe shading (*−*14% in biomass and −37% in HI).

### Effects of shading on grain protein accumulation and gluten composition, and straw N content

The grain protein content increased considerably with shading (Table 2). In *Bologna* (second year), protein increases were +1.7% with moderate shading and +1.9% with severe shading conditions. Maximum increases were observed in older varieties under moderate shade (+1.7% for *Piave* and +3.4% for *Terminillo*; absolute variations).

**Table 2:** Grain protein content and composition, and straw nitrogen (means ± SEs; *n* = 3) for *Bologna* (2018–2019 and 2019–2020), *Piave* and *Terminillo* (2018–2019 only), under two shading treatments (−30 and −50% PAR) compared with full sun controls (C).

Variety	Year	Treatment	Grain proteins (% DW)		Gluten (mg g^−1^)		Gliadins (mg g^−1^)		HMW‐GS (mg g^−1^)		LMW‐GS (mg g^−1^)		Glutenins‐to‐Gliadins ratio		Straw nitrogen content (% DW)	
*Bologna*	2018–2019	C	13.9 ± 0.9 b	Ref.	29.4 ± 1.1 b	Ref.	20.3 ± 0.9 b	Ref.	4.2 ± 0.2 b	Ref.	4.9 ± 0.2a	Ref.	0.45 ± 0.01a	Ref.	0.84 ± 0.11 b	Ref.
		−30% PAR	14.6 ± 0.4 a	(+0.7)	32.1 ± 0.0 a	(+9%)	22.4 ± 0.2 a	(+11%)	5.0 ± 0.3 a	(+20%)	4.6 ± 0.2 a	(−6%)	0.43 ± 0.01 a	(−4%)	0.88 ± 0.09 ab	(+5%)
		−50% PAR	15.7 ± 0.1 a	(+1.8)	33.2 ± 1.0 a	(+13%)	23.5 ± 0.9 a	(+16%)	5.0 ± 0.3 a	(+20%)	4.7 ± 0.3 a	(−5%)	0.42 ± 0.01 a	(−8%)	1.46 ± 0.18 a	(+75%)
	2019–2020	C	12.9 ± 0.5 b	Ref.	32.9 ± 1.0 b	Ref.	23.1 ± 0.6 b	Ref.	4.3 ± 0.3 a	Ref.	5.5 ± 0.1a	Ref.	0.43 ± 0.01a	Ref.	0.90 ± 0.15 a	Ref.
		−30% PAR	14.6 ± 0.9 a	(+1.7)	34.6 ± 0.4 a	(+5%)	24.3 ± 0.1 a	(+5%)	4.6 ± 0.3 a	(+7%)	5.6 ± 0.2 a	(+2%)	0.42 ± 0.02 a	(−1%)	0.83 ± 0.17 a	(−7%)
		−50% PAR	14.8 ± 0.7 a	(+1.9)	34.4 ± 0.2 a	(+5%)	24.3 ± 0.2 a	(+5%)	4.6 ± 0.3 a	(+7%)	5.4 ± 0.1 a	(−1%)	0.42 ± 0.02 a	(−2%)	0.78 ± 0.09 a	(−13%)
*Piave*	2018–2019	C	15.0 ± 0.3 b	Ref.	38.6 ± 1.4 c	Ref.	29.9 ± 1.6 a	Ref.	3.5 ± 0.2 b	Ref.	5.3 ± 0.1 a	Ref.	0.30 ± 0.02 a	Ref.	1.30 ± 0.11 a	Ref.
		−30% PAR	16.7 ± 0.7 a	(+1.7)	45.4 ± 1.5 a	(+18%)	35.0 ± 1.3 a	(+17%)	5.1 ± 0.1 a	(+48%)	5.3 ± 0.1 a	(+1%)	0.30 ± 0.01 a	(=)	1.34 ± 0.11 a	(+3%)
		−50% PAR	16.4 ± 0.7 a	(+1.4)	41.4 ± 0.7 b	(+7%)	31.8 ± 0.7 a	(+7%)	4.1 ± 0.4 ab	(+20%)	5.5 ± 0.2 a	(+2%)	0.30 ± 0.01 a	(−1%)	1.27 ± 0.05 a	(−2%)
*Terminillo*	2018–2019	C	15.8 ± 0.3 b	Ref.	49.1 ± 0.4 c	Ref.	37.3 ± 0.4 b	Ref.	6.0 ± 0.1 a	Ref.	5.9 ± 0.2 a	Ref.	0.32 ± 0.01 a	Ref.	1.29 ± 0.08 a	Ref.
		−30% PAR	19.2 ± 1.5 a	(+3.4)	54.2 ± 0.6 a	(+10%)	41.4 ± 0.2 a	(+11%)	6.7 ± 0.3 a	(+12%)	6.0 ± 0.1 a	(−3%)	0.31 ± 0.01 a	(−3%)	1.26 ± 0.09 a	(−2%)
		−50% PAR	17.3 ± 0.5 a	(+1.4)	51.6 ± 0.6 b	(+5%)	39.6 ± 0.7 a	(+6%)	6.0 ± 0.2 a	(=)	6.0 ± 0.2 a	(−5%)	0.30 ± 0.01 a	(−5%)	1.27 ± 0.08 a	(−2%)

	Year	Factor	Significance levels													
ANOVA	2018–2019	Variety	**		***		***		***		***		***		*	
		Shading	*		***		***		***		n.s.		n.s.		n.s.	
		Variety × Shading	n.s.		n.s.		n.s.		n.s.		n.s.		n.s.		*	
	2019–2020	Shading	*		*		*		n.s.		n.s.		n.s.		n.s.	

For each parameter, different letters indicate statistically significant differences among treatments (Tukey's HSD test, *P* ≤ 0.05) within each variety and year. Values in parentheses indicate the absolute variation for grain proteins and the percentage variation of treatments relative to the control (Ref.) for the other parameters. Results of ANOVA are reported in the bottom block, with significance levels for the main effects (variety and shading) and their interaction (n.s. = not significant; *, ** and *** indicate significance at *P* ≤ 0.05, *P* ≤ 0.01, and *P* ≤ 0.001, respectively).

Abbreviation: HMW‐GS, high‐molecular‐weight glutenin subunits; LMW‐GS, low‐molecular‐weight glutenin subunits; PAR, photosynthetic active radiation.

The gluten content followed a similar pattern: in *Bologna* it increased by +9% under moderate shading and +13% under severe shading conditions in the first year, with a uniform +5% rise in the second year. *Piave* and *Terminillo* had the highest gluten levels under moderate shade (45.4 and 54.2 mg g^−1^, respectively) with +18 and +10% increases respectively (*P* ≤ 0.05).

Increases in gluten content were mainly associated with higher gliadin levels across years and varieties. In the first year, gliadin content increased under shading by 13% in the *Bologna* and *Piave* varieties, and by 8% in *Terminillo* compared with controls (Table 2).

Glutenin content changes were more variable. The *Bologna* variety showed modest increases (+3% in the first year and +4% in the second year compared with the respective controls; *P* ≥ 0.05). This was mainly driven by increases in HMW‐GS subunits (+20% in the first year and +7% in the second year under both shading intensities compared with controls; *P* ≤ 0.05). In the two old varieties under moderate shading, glutenin increases were more marked than in the *Bologna* variety, with *Piave* and *Terminillo* increasing by 19% and 8% respectively, relative to controls. These increases were largely driven by higher HMW‐GS content, with statistically significant changes observed in *Piave*.

The glutenin/gliadin ratio showed a consistent, although non‐significant, downward trend in both seasons and all the varieties.

Straw N content in the Bologna variety showed considerable variation, with a 75% increase compared with the control under severe shading conditions in the first year (*P* ≤ 0.05) with minor reductions in the second year. In contrast, the older varieties exhibited only slight reductions in straw N content under severe shading.

### Mineral composition of grains under shading compared with full sun

**Figure 4:**
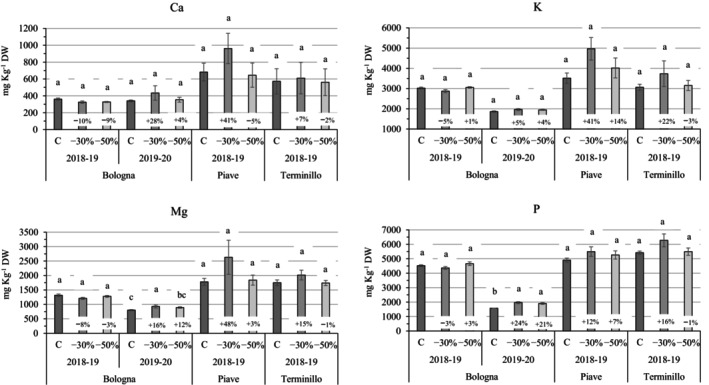
Mineral concentration in grains for *Bologna* (2018–2019 and 2019–2020), *Piave* and *Terminillo* (2018–2019 only), under two artificial shading treatments (−30 and −50% reduction in photosynthetic active radiation (PAR)) vs. full sun controls (C). Within each variety and year, the numbers in the histograms indicate the percentage variation relative to the control, and different letters indicate statistically significant differences among treatments (Tukey's HSD test, *P* ≤ 0.05).

The two old varieties generally showed higher concentrations of minerals in the grains than the *Bologna* variety (approximately +70% K, Mg, and P, and +35% Ca) (Fig. 4; Supporting Information, Table SI.3). In the *Bologna* variety, the effect of shading varied by year; in the first year, shading resulted in slight and non‐significant decreases in Ca and Mg (−10 and −5% vs C), whereas in the second year all the investigated minerals increased under shading, particularly Mg and P, which increased by 14% and 22%, respectively. In old varieties moderate shading generally improved grain mineral concentrations (up to +48% for Mg in the *Piave* variety in the first year), although not significantly. Under severe shading conditions, only K in the *Piave* variety was observed to improve slightly (+14% vs C), although not significantly.

### Summarizing results by principal component and discriminant analyses

**Figure 5:**
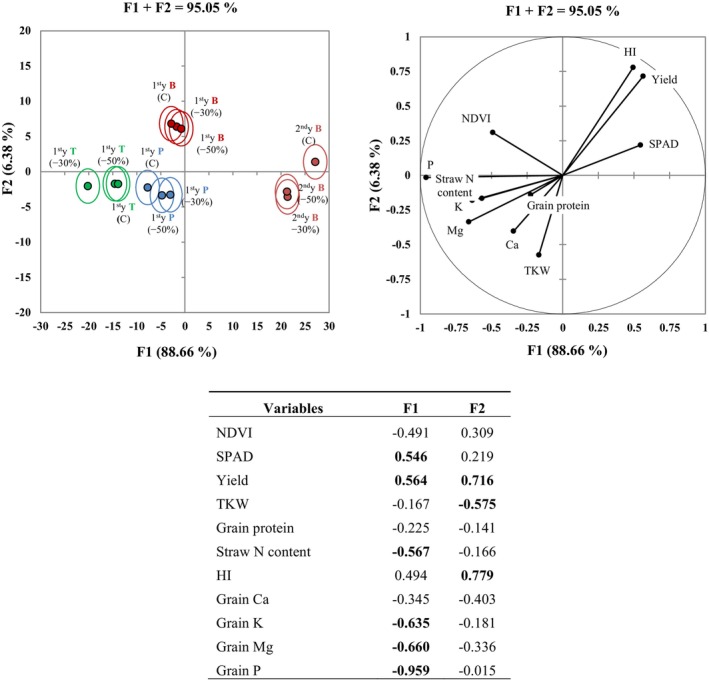
Multigroup discriminant analysis (MDA; left) and principal component analysis (PCA; right) for vegetation indices, yield, and quality parameters, and grain mineral concentrations for *Bologna* (2018–2019 and 2019–2020) *Piave*, and *Terminillo* (2018–2019 only), under two shading treatments (−30 and −50% PAR) vs. full sun controls (C). The isodensity confidence circles represent 75% variability. In the lower table, the highly informative variables (loadings > |0.5|) are highlighted in bold, within synthetic variables F1 and F2.

Principal component analysis (PCA) identified two synthetic variables (F1 and F2), which explained approximately 89% and 6% of the overall variability, respectively (Fig. 5). Variables with loadings greater than |0.5| in F1 included leaf chlorophyll content (SPAD), grain yield, TKW and HI, straw N content, and grain mineral concentrations of K, Mg, and P. Multigroup discriminant analysis (MDA) further revealed that the effects of shading in *Piave* and *Terminillo* were mostly associated with variations in TKW and grain mineral composition (P, Mg, Ca, and K). In the *Bologna* variety, however, the shading effects were mostly linked to variations in yield, HI, and to the leaf greenness (SPAD), regardless of the year of cultivation. Notably, under full sun controls the *Bologna* variety produced a better yield in the second year than the first, but at the expense of grain quality. In the first year, the shading treatments in *Bologna* and *Piave* were similar to full sun controls, but this was not the case in *Terminillo*. In the second year, the *Bologna* variety exhibited a clear separation between controls and shade treatments, irrespective of shade intensity.

## DISCUSSION

This study offers new insights into how shading affects wheat physiology, development, and yield depending on the varietal traits and the intensity of solar radiation reduction under two contrasting growing seasons in terms of weather conditions. When PAR is reduced, the first observable effect is a delay in wheat phenology accompanied by some physiological and morphological adaptations. Although the shading was applied in late April during wheat stem elongation, it delayed heading and flowering significantly in all varieties, with the effect being almost proportional to shading intensity only in *Bologna*. Prolonged stay‐green and photosynthetic activity, with delayed senescence of the flag leaf, were also observed in all the investigated wheat varieties under shading. An increased leaf area duration is a crucial plant adaptation to shading, which has been documented to positively affect grain yield as well as the kernel size and quality.^40^, ^41^, ^42^, ^43^, ^44^, ^45^ As noted above, plants under different shading treatments may have been at slightly different developmental stages due to shading‐induced phenological delay. Consequently, asynchrony in physiological metrics such as NDVI, chlorophyll content, and leaf‐area duration may reflect both the direct impact of shading and a minimal indirect effect of delayed phenology.^46^

Phenological responses to shading vary with regional climate and irradiance. In the Mediterranean climate of southern France, Inurreta‐Aguirre *et al*.^20^ reported an approximate 1 week delay in wheat flowering when intercropped with 16‐year‐old poplars – consistent with the findings of the current study – whereas Dufour *et al*.^15^ observed no significant phenological changes under 42% to 60% artificial light reduction. By contrast, in the continental climate of central Spain, Arenas‐Corraliza *et al*.^24^ recorded an advancement of early phenological stages (up to the onset of milk development) in wheat and barley when subjected to 25% to 50% PAR reduction. They suggested that such advancement may mitigate heat stress during later stages in these high‐irradiance zones. Indeed, central Spain receives approximately 6300 MJ m^−2^ year^−1^ of cumulative solar radiation, compared with 5446 MJ m^−2^ year^−1^ in southern France and 5322 MJ m^−2^ year^−1^ in northeastern Italy.^47^ These geographical variations in light availability and thermal regimes appear to be essential in determining whether shading promotes or delays crop development.

Delayed leaf senescence is commonly associated with shading,^24^, ^48^ although a positive contribution to wheat yield has been clearly demonstrated only under mild shading (*−*12% PAR) applied after anthesis.^22^ Indeed, this study demonstrates that the *Bologna* variety had an 8% yield gain in the first (rainy) year under 30% shading applied from late stem elongation until harvest, along with slight improvements in TKW and HI. In contrast, many studies reported marked yield losses under shading,^49^, ^50^, ^51^ although the impact varies according to shading intensity and time of application. With a moderate PAR reduction of about *−*30%, as in the current study, yield impairments can range from −20% to −50%.^15^, ^23^, ^52^ Under severe shading conditions (*−*43% to *−*66% of PAR), documented wheat yield losses vary from *−*25% to *−*80% compared to full sun controls.^14^, ^53^, ^54^ The current study confirmed marked yield reduction in the old wheat varieties *Terminillo* and, particularly, in *Piave* (*−*66%) under both moderate and severe shading. Unexpectedly, the *Bologna* variety also exhibited significant grain yield losses in the second (dry) year, ranging from *−*38% at *−*30% PAR to *−*55% at *−*50% PAR.

It is acknowledged that direct cultivar comparisons across years are constrained by environmental contrasts; furthermore, genotype × environment studies necessitate multi‐year trials to address shade‐tolerance variability. However, these results should be interpreted in light of the specific weather conditions experienced in the 2 years in which the experiment was conducted. The second year was notably drier and warmer than the first (Supporting Information, Fig. SI.1). Radiation availability during the key March–June period also declined by *−*6.8% in the second year relative to the first one, primarily due to lower values in April and May (Supporting Information, Fig. SI.2). This combination of weather conditions likely intensified the impact of shading, particularly during grain filling, thus emphasizing how annual variations in weather, especially solar radiation during critical growth stages, can strongly influence the outcomes of shading treatments in wheat.

The results of this study point to solar radiation as the main driver of the observed responses but it is important to note that in mature agroforestry systems, rain interception by tree canopies can further reduce soil water availability to understory crops – a phenomenon described by Bachakdjian *et al*.^55^ in their work on adapting rainout shelters to agroforestry systems. However, to better understand the water balance effects in shaded agroecosystems, future studies should incorporate direct measurements of soil moisture and crop water use along with evapotranspiration. The old tall varieties (122 cm in height versus 88 cm in the *Bologna* variety) were strongly affected by abundant rainfall and lodging in spring 2019 (the first year), which impacted their yield under shading nets. In this way modern varieties may better tolerate both shading and drought, whereas old varieties appear less suitable for agroforestry systems with variable water conditions, although windbreak and shelter effects from trees may mitigate some adverse climatic impacts.^56^ The pronounced yield loss under shade observed in the second year in the high‐yielding *Bologna* variety is likely due to the severe drought stress during the growing period from tillering to grain filling (134 mm from January to May versus the 335 mm historical mean). Low water availability post‐anthesis is widely recognized as a primary cause of yield reduction in wheat.^57^, ^58^

In agroforestry systems with frequent and abundant rainfall, crop productivity under shading is generally limited by above‐ground competition for solar radiation.^59^, ^60^, ^61^, ^62^ In contrast, during heat waves, periods of low rainfall during spring/summer, productivity may be limited by both solar radiation and water availability.^63^, ^64^, ^65^, ^66^

Yield components are affected differently by reduced irradiance, depending on the time of shading. Pre‐flowering shading is mainly associated with reductions in the number of grains per unit surface area, due to fewer numbers of kernels per spike.^67^, ^68^, ^69^, ^70^ In contrast, shading from flowering to maturity typically has greater negative impact on both the number of grains per square meter and grain weight.^14^, ^71^ Although not measured in this study, the number of grains per spike is often considered the most affected yield component.^15^, ^20^, ^72^ Here, TKW was somewhat reduced in the old *Piave* variety, which, despite having the greatest kernel weight, suffered the most substantial yield losses, while it rarely increased in the *Bologna* variety. However, the most impacted yield component was HI, particularly in the old varieties and in *Bologna* during the second year. This reduction in HI is attributable to enhanced vegetative growth under shading, in agreement with Dos Santos *et al*.,^56^ who reported increased LAI‐to‐CAI ratio, plant height, and biomass, especially in old wheat varieties under severe shading within a poplar plantation.

Previous studies^21^, ^23^ have shown that, when applied from the jointing stage to maturity, moderate shading – corresponding to an 8% to 22% reduction in PAR – generally leads to only minor decreases in grain yield, highlighting its potential for sustainable crop management in agroforestry systems. Moreover, when similar shading is applied post‐anthesis, slight yield increases have been reported.^21^, ^22^ In high‐irradiance environments, moderate shading may even enhance yield.^18^, ^19^ Yield improvements similar to those observed in the *Bologna* variety during the first year of the experiment have also been recorded in alley‐cropping systems with young trees in temperate climates.^17^

Low shading intensity may promote morphological and physiological adjustments that enhance light interception and radiation use efficiency, although it seldom translates into grain yield. Increased leaf chlorophyll content is commonly associated with shade tolerance, improving light‐harvesting and photosynthetic efficiency.^65^, ^73^, ^74^ This study confirms that the flag leaf chlorophyll content (SPAD index) increased with shading in the *Bologna* variety and was significantly higher in *Piave*. Several studies have reported an increase in the chlorophyll content of the flag leaf under various levels of light reduction, highlighting its key role in shading acclimation and maintenance of high photosynthetic rate.^21^, ^75^, ^76^

Another strategy for optimizing light interception under shading is a change in crop canopy architecture. In this trial, the *Bologna* variety exhibited some variations in canopy structure, although not significantly, possibly due to the late application of artificial shading, as previously suggested by Artru *et al*.^19^ Specifically, LAI was slightly reduced while the LAI‐to‐CAI ratio increased, suggesting a compensatory mechanism to improve light interception, as reported by Dos Santos *et al*.^56^ Mu *et al*.^28^ noted that reductions in LAI under shading can be partially offset by an increased top‐to‐bottom leaf area ratio. In some studies, shading adaptation has been associated with increased leaf area, a typical shade‐avoidance strategy. However, this response often reduces yield, as more resources are diverted to leaf and stem growth at the expense of grain production.^20^, ^23^, ^24^ An increase in plant height is also commonly observed in shaded environments, mainly due to the elongation of the last internode.^19^, ^21^, ^77^ A similar trend was observed in the *Bologna* variety, although it was only a trend. However, measuring these morphological traits in the two old varieties would have clarified the potential genetic variability in shading acclimation, recent research has shown contrasting responses in plant height among modern wheat varieties, with a generally marked increase in the high‐sized old varieties.^54^ The morphological changes under shading in this study, notably the tendency towards increasing spike length and final internode length, may be a compensatory response in biomass allocation. This aligns with the findings of Abbate *et al*.,^78^ who found higher biomass partitioning to the ear in wheat under shaded conditions. That response shows that, under reduced radiation, wheat may prioritize assimilate allocation to reproductive organs, possibly as an adaptation strategy to sustain reproductive success despite lower irradiance.

The effects of shading on grain quality remain relatively under‐investigated, yet this study highlights a significant improvement in grain protein content. A concentration effect was confirmed, as the most relevant enhancement of protein content was associated with the strongest yield reduction.^14^, ^24^, ^79^ Higher protein rates in wheat grains are often reported under artificial shading^14^, ^15^, ^19^ as well as in silvoarable systems adjacent to tree rows.^17^, ^45^, ^72^ In this study, the innovative investigation into the effects of shading on gluten content and composition revealed that shading sustained increased gluten accumulation – particularly in the *Bologna* variety and *Terminillo* – primarily due to an increase in gliadins rather than glutenins, which may enhance dough extensibility.^72^, ^80^, ^81^ The distribution pattern of glutenins in response to shading has been linked to the regulation of HMW‐glutenin subunits (HMW‐GS).^40^ Indeed, in our study, HMW‐GS increased by up to +48% in the old *Piave* variety, suggesting improved dough tenacity.

Altered evapotranspiration and carbon partitioning under shading may also affect plant nutrition. This study provides new insights into the translocation of Ca, Mg, K, and P – nutrients that are typically low in cereal grains. The two old wheat varieties were richer in minerals than *Bologna* and showed further improvement under moderate shading. In *Bologna*, shading effects on mineral content were evident in the second, drier year – particularly for Mg and P – likely as a consequence of reduced yield.

In mature agroforestry systems, shading nets are increasingly used to study the effects of shade on field crops, as they are inexpensive, easy to apply, and provide consistent irradiance reduction.^14^ However, these do not fully replicate the complex and dynamic light environment seen beneath actual tree canopies. In agroforestry systems, shading intensity develops progressively during leaf development of trees and is highly variable, being influenced by canopy architecture, diurnal solar elevation angle, seasonal dynamics and varied shade patterns resulting from wind‐driven canopy movements.^82^, ^83^, ^84^

In real tree–crop environments, brief sunflecks – transient bursts of full sunlight alternating with shade – can contribute over 30% of the total daily photon flux and substantially influence crop carbon gain.^85^ Recent findings by Acevedo‐Siaca *et al*.^86^ show that cultivated rice (*Oryza sativa*) exhibits slower photosynthetic induction and reduced cumulative CO_2_ uptake in comparison with its wild relatives. These differences are particularly evident under dynamic light conditions that mimic natural field environments. These results underscore a key limitation of using constant‐shade nets, which are not able to completely capture the dynamic effects of intermittent light of agroforestry systems.

In modern, low‐density alley silvoarable systems – with approximately 30–50 trees per hectare – the average light reduction across the inter‐row is likely lower than the moderate shading (−30%) tested in this study. There is, therefore, significant potential for successfully incorporating wheat into agroforestry systems in temperate climates, potentially leading to efficient acclimation and even yield benefits under climate‐change scenarios.

## CONCLUSIONS

Wheat adaptation to shading is feasible, as demonstrated by a range of plastic morpho‐physiological responses, including increased leaf chlorophyll content, enhanced canopy greenness, and delayed senescence, as well as some minor improvements in LAI, the LAI‐CAI ratio, and plant height. Although grain yield responses vary depending on variety choice and climatic conditions/year, the improvements in grain protein content and mineral concentrations are valuable effects of shading.

Further validation in actual agroforestry systems is required; however, these results indicate that mature modern agroforestry designs – with low tree‐density and consequently milder PAR reduction than the −30% tested here may potentially achieve better yield outcomes. It is also essential to expand varietal screening to identify suitable genotypes for each environment and to pinpoint specific morpho‐physiological traits that can guide breeding programs. In the meantime, modern varieties appear more promising for agroforestry applications than old local tall ones, as their productivity remains considerably higher, even under severe shading conditions. Nevertheless, old varieties maintained in local germplasm banks, represent crucial genetic resources that could be exploited under extremely severe shading conditions, such as those encountered in agroforestry settings with evergreen tree species, multi‐strata hedgerows and design with higher tree densities, or even in challenging agrivoltaics systems. To accelerate research in this field, future investigations should compare different artificial shading systems (e.g. nets vs. slats) to mimic the dynamic lighting conditions in agroforestry systems.

## AUTHOR CONTRIBUTIONS

Anna Panozzo: conceptualization, methodology, formal analysis, investigation, data curation, writing – original draft preparation, writing – review and editing, visualization. Pranay Kumar Bolla: writing – review and editing. Giuseppe Barion: investigation. Giovanna Visioli: methodology. Teofilo Vamerali: conceptualization, methodology, resources, writing – review and editing, supervision.

## FUNDING INFORMATION

This work received no funding.

## CONFLICT OF INTEREST

The authors declare no conflict of interest.

## Supporting information


**Data S1.** Supporting Information.

## Data Availability

The data that support the findings of this study are available from the corresponding author upon reasonable request.
