# Supplementary material for: Morpho‐physiological and agronomic responses of wheat varieties under artificial shade in agroforestry systems
Source: J Sci Food Agric. 2025 Sep 4;106(1):303–16. doi: 10.1002/jsfa.70157 (PMC12623277; doi:10.1002/jsfa.70157)
Supplement: Supplementary file 1 — Data S1. Supporting Information. [file JSFA-106-303-s001.docx]

SUPPLEMENTARY INFORMATION

**Figure SI.1.** Monthly mean temperatures and rainfall during the wheat cycle in 2018-2019 and 2019-2020 compared to the 10-year historical mean (2008-2018 and 2009-2019) at Legnaro experimental site (Padua, NE Italy).

**Figure SI.2.** Monthly average daily global radiation (MJ m⁻² day⁻¹) at the Legnaro experimental site during October–June of the 2018–19 (left) and 2019–20 (right) wheat growing seasons. Gray bars indicate observed monthly means, and the black line represents the 10‑year historical mean (2008-2018 and 2009-2019). Values above each bar denote the exact monthly radiation.

**Figure SI.3.** Photographs of wheat during grain maturation on 8 June (both years) in plots of wheat varieties Bologna (2018–19 and 2019–20) and Piave and Terminillo (2018–19 only), under two artificial shading treatments (–30% and –50 PAR) vs. full sun controls (C).

**Table SI.1.** Results of ANOVA for the NDVI (average of all the measurements from the last week of April to maturity in June) and SPAD (measured during the last week of May) indices for wheat var. Bologna (2018–19 and 2019–20) and Piave and Terminillo (2018–19 only), under two artificial shading treatments (–30% and –50% PAR) vs. full sun controls (C). Significance for the main effects (Variety and Shading) and their interaction (n.s. = not significant; *, ** and *** = significance at *P* ≤ 0.05, *P* ≤ 0.01 and *P* ≤ 0.001, respectively).

|  |  |  | NDVI | SPAD |
| --- | --- | --- | --- | --- |
| 2018-19 | Variety |  | * | *** |
|  | Shading |  | ns | ns |
|  | Variety × Shading |  | ns | * |
| 2019-20 | Shading |  | * | ns |

**Table SI.2**. Morphological traits (mean ± S.E.; n = 3) of wheat variety Bologna during the 2^nd^ year (2019–20), under two shading treatments (–30% and –50% PAR) vs. full sun controls (C). LAI: leaf area index; CAI: culm area index. For each parameter, letters indicate significant differences among treatments (Tukey’s HSD test, p ≤ 0.05), and the values in brackets represent the percentage variation of the shading treatment relative to C. Results of ANOVA are reported below, with significance for the main effects Shading (n.s. = not significant; *, ** and *** = significance at p ≤ 0.05, p ≤ 0.01 and p ≤ 0.001, respectively).

| **Variety** | **Treatment** |  | **LAI** | |  | **LAI/CAI** | |  | **Plant height**  **(cm)** | |  | **Spike length**  **(cm)** | | **Last internode length**  **(cm)** | |
| --- | --- | --- | --- | --- | --- | --- | --- | --- | --- | --- | --- | --- | --- | --- | --- |
| Bologna | C |  | 4.9±0.9 a | Ref. |  | 2.0±0.4 a | Ref. |  | 85.3±1.1 a | Ref. |  | 7.5±0.3 a | Ref. | 11.7±0.7 a | Ref. |
|  | *–*30% |  | 4.5±0.8 a | (*–*9) |  | 2.1±0.4 a | (+6) |  | 83.7±1.4 a | (*–*2) |  | 7.4±0.3 a | (*–*1) | 12.4±0.8 a | (+7) |
|  | *–*50% |  | 4.2±0.7 a | (*–*15) |  | 2.2±0.4 a | (+11) |  | 85.7±1.6 a | (=) |  | 7.9±0.3 a | (+5) | 12.1±0.7 a | (+4) |
| 2019-20 | Shading |  | ns |  |  | ns |  |  | ns |  |  | ns |  | ns |  |

**Table SI.3.** Results of ANOVA for the mineral concentration in grains for wheat var. Bologna (2018–19 and 2019–20) and Piave and Terminillo (2018–19 only), under two artificial shading treatments (–30% and –50% PAR) vs. full sun controls (C). Significance for the main effects (Variety and Shading) and their interaction (n.s. = not significant; *, ** and *** = significance at *P* ≤ 0.05, *P* ≤ 0.01 and *P* ≤ 0.001, respectively).

|  |  |  | Ca |  | K |  | Mg |  | P |
| --- | --- | --- | --- | --- | --- | --- | --- | --- | --- |
| 2018-19 | Variety |  | ** |  | ** |  | *** |  | *** |
|  | Shading |  | ns |  | ns |  | ns |  | ns |
|  | Variety × Shading |  | ns |  | ns |  | ns |  | ns |
| 2019-20 | Shading |  | ns |  | ns |  | * |  | ** |
